# Supplementary material for: Identifying predictors of formal help-seeking for premenstrual symptoms: A machine learning analysis of symptom, functional impairment and barriers data
Source: PLOS Ment Health. 2025 Aug 27;2(8):e0000274. doi: 10.1371/journal.pmen.0000274 (PMC12798355; doi:10.1371/journal.pmen.0000274)
Supplement: S1 Data — (DOCX) [file pmen.0000274.s001.docx]

**S1 Appendix. Summary of sociodemographic characteristics***.*

|  | **Overall (N=592)** | | | **Previous help-seeker (n=339)** | | | **Non-help-seeker (n=253)** | | |  |  |  |  |
| --- | --- | --- | --- | --- | --- | --- | --- | --- | --- | --- | --- | --- | --- |
|  | **n** | **%** | **Mean (SD, range)** | **n** | **%** | **Mean (SD, range)** | **n** | **%** | **Mean (SD, range)** | ***X* ^2^ (df)** | ***U*** | **P** | **Φc** |
| **Age** |  |  | 34 (18-51) |  |  | 35 (18-50) |  |  | 33 (18-51) |  | 36189.00 | .001 |  |
| **Gender** | | | | | | | | | | | | | |
| Woman | 576 | 97.30 |  | 332 | 97.94 |  | 244 | 96.44 |  | 3.194 |  | .272 | .075 |
| Non-binary | 14 | 2.36 |  | 6 | 1.77 |  | 8 | 3.16 |  |  |  |  |  |
| Other | 1 | 0.17 |  | 0 | 0.00 |  | 1 | 0.40 |  |  |  |  |  |
| Prefer not to answer | 1 | 0.17 |  | 1 | 0.29 |  | 0 | 0.00 |  |  |  |  |  |
| **Ethic group** | | | | | | | | | | | | | |
| White or Caucasian | 544 | 91.89 |  | 311 | 91.74 |  | 233 | 92.09 |  | 3.832 |  | .761 | .084 |
| Asian (Indian, Pakistani, Bangladeshi, Chinese, or any other Asian background) | 18 | 3.04 |  | 8 | 2.36 |  | 10 | 3.95 |  |  |  |  |  |
| Black, Caribbean or African | 4 | 0.68 |  | 2 | 0.59 |  | 2 | 0.79 |  |  |  |  |  |
| Hispanic or Latinx | 6 | 1.01 |  | 4 | 1.18 |  | 2 | 0.79 |  |  |  |  |  |
| Mixed or multiple ethnic groups | 17 | 2.87 |  | 11 | 3.24 |  | 6 | 2.37 |  |  |  |  |  |
| Other ethnic group | 2 | 0.34 |  | 2 | 0.59 |  | 0 | 0.00 |  |  |  |  |  |
| Prefer not to answer | 1 | 0.17 |  | 1 | 0.29 |  | 0 | 0.00 |  |  |  |  |  |

|  | **Overall (N=592)** | | | **Previous help-seeker (n=339)** | | | **Non-help-seeker (n=253)** | | |  |  | | |  |  |
| --- | --- | --- | --- | --- | --- | --- | --- | --- | --- | --- | --- | --- | --- | --- | --- |
|  | **n** | **%** | **Median (range)** | **n** | **%** | **Median (range)** | **n** | **%** | **Median (range)** | ***X* ^2^ (df)** | ***U*** | | | **P** | **Φc** |
| **Highest educational attainment** | | | | | | | | | | | | | | | |
| Primary education or below^a^ | 1 | 0.17 |  | 1 | 0.29 |  | 0 | 0.00 |  | 2.628 | |  | .942 | | .069 |
| Lower secondary education^b^ | 16 | 2.70 |  | 10 | 2.95 |  | 6 | 2.37 |  |  |  |  |  |  |  |
| Upper secondary education^c^ | 75 | 12.67 |  | 42 | 12.39 |  | 33 | 13.04 |  |  |  |  |  |  |  |
| Undergraduate degree | 241 | 40.71 |  | 134 | 39.53 |  | 107 | 42.29 |  |  |  |  |  |  |  |
| Postgraduate degree | 245 | 41.39 |  | 142 | 41.89 |  | 103 | 40.71 |  |  |  |  |  |  |  |
| Other | 13 | 2.20 |  | 9 | 2.65 |  | 4 | 1.58 |  |  |  |  |  |  |  |
| Prefer not to answer | 1 | 0.17 |  | 1 | 0.29 |  | 0 | 0.00 |  |  |  |  |  |  |  |
| **Employment*** | | | | | | | | | | | | | | | |
| Employed^d^ | 494 | 83.45 |  | 285 | 84.07 |  | 209 | 82.61 |  | .224 (1) | |  | .656 | | .019 |
| Unemployed | 28 | 4.73 |  | 16 | 4.72 |  | 12 | 4.74 |  | .000 (1) | |  | 1.000 | | .001 |
| Student | 74 | 12.50 |  | 33 | 9.73 |  | 41 | 16.21 |  | 5.547 (1) | |  | .019 | | .097 |
| Homemaker | 31 | 5.24 |  | 23 | 6.78 |  | 8 | 3.16 |  | 3.831 (1) | |  | .050 | | .080 |
| Maternity/paternity leave or taking time off of work to care for a family member | 13 | 2.20 |  | 8 | 2.36 |  | 5 | 1.98 |  | .099 (1) | |  | .753 | | .013 |
| Voluntary work | 17 | 2.87 |  | 5 | 1.47 |  | 12 | 4.74 |  | 5.548 (1) | |  | .019 | | .097 |
| Prefer not to answer | 2 | 0.34 |  | 2 | 0.59 |  | 0 | 0.00 |  | N/A | |  | N/A | | N/A |

*Key. * Percentage total may exceed 100 as participants were able to select multiple answer options. a: up to 11 years; b: up to 16 years; c: up to 18 years; d: includes employed full-time, employed part-time and self-employed*
