# Supplementary material for: Identifying predictors of formal help-seeking for premenstrual symptoms: A machine learning analysis of symptom, functional impairment and barriers data
Source: PLOS Ment Health. 2025 Aug 27;2(8):e0000274. doi: 10.1371/journal.pmen.0000274 (PMC12798355; doi:10.1371/journal.pmen.0000274)
Supplement: Attachment — Submitted filename: Response letter JTSBEFrev.docx [file pmen.0000274.s003.docx]

**PLOS Mental Health**

19th May 2025

Dear Editorial Board,

**Re: Erin L. Funnell, Nayra A. Martin-Key, Jakub Tomasik & Sabine Bahn: Identifying Predictors of Formal Help-Seeking for Premenstrual Symptoms: a Machine Learning Analysis of Symptom, Functional Impairment and Barriers Data**

Thank you for giving us the opportunity to revise our manuscript for consideration by *PLOS Mental Health*. We would like to sincerely thank the reviewers for providing feedback on our manuscript. We hope that we have addressed the reviewers’ comments to their satisfaction.

To summarise the amendments in the revised manuscript, we have expanded the methods section to provide more details about the model tuning and testing process. We have also revised the limitations section to add further discussion about the sample limitations and methodological recommendations for future similar works. We also ran LASSO logistic regression model analysis in addition to the XGBoost model, which is presented and discussed in the reviewers replies.

We have provided a reply or rebuttal of each of the comments made by reviewers and detailed where any relevant amendments to the manuscript have been made.

We hope that the revised manuscript is now acceptable for publication in *PLOS Mental Health.* We look forward to hearing from you at your earliest convenience.

Yours sincerely,

Professor Sabine Bahn MD PhD MRCPsych FRSB, signed on behalf of all co-authors
Director of Cambridge Centre for Neuropsychiatric Research
Professor of Neurotechnology
Department of Chemical Engineering and Biotechnology
University of Cambridge
Philippa Fawcett Drive
Cambridge CB3 0AS, UK
Tel +44 1223 334151
Fax +44 1223 334162
<http://ccnr.ceb.cam.ac.uk/>

e-mail [sb209@cam.ac.uk](mailto:sb209@cam.ac.uk)

**Reviewer #1**

This manuscript was well written and the machine learning strategy is certainly a technique matching the objectives set out by the authors. The conclusions followed from the analyses in that the current study identified predictors of formal help-seeking for premenstrual symptoms, possibly pointing to the need for improved education to improve help-seeking behaviors. The limitations were duly noted by the investigators. However, despite the limitations, the information was processed and presented successfully.

**Thank you very much for taking the time to review our manuscript, and for your feedback. We hope that we have addressed each of the points raised to your satisfaction.**

The sample size was certainly adequate. The XGBoost algorithm was properly selected due to its capabilities in detecting non-linear relationships and interactions between variables, being robust to correlated and non-normally distributed features. Model performance and validation were conducted. The AOC was reasonable. It would have been helpful to have a bit more detail such as size of training and test sets and the performance of the confusion matrix in the process, unless this reviewer missed something.

**Thank you for this suggestion. We now clarify the size of the training and test sets in the Results Section (lines 200-201). We also provide further confusion matrix performance metrics – such as the positive and negative predictive values – in the Results Section (lines 197-200).**

Statistically, the prediction performance was well explained and the comparison of the help-seekers and non-help-seekers using the Mann-Whitney U tests clearly indicated (Table1) that there was a significant difference in the presence and severity of all symptoms and domains of functional impairment.

**Thank you for this summary and for your review.**

**Reviewer #2**

This paper addresses an important and highly relevant topic related to women's wellbeing, contributing to the de-stigmatization of conversations around menstrual symptoms and investigating the barriers and predictors of help-seeking behavior.

**Thank you very much for taking the time to review our manuscript. We appreciate your feedback and hope that we have sufficiently addressed each suggested amendment.**

The paper is well-written, with a particularly strong introduction and discussion of both the benefits and barriers to seeking help. The sample size is relatively large (592 participants in total, 339 of whom sought help), covering a broad age range (18–55).

The methodology appears to be a weaker aspect of the study:

Firstly, while XGBoost is a powerful algorithm, research suggests that logistic regression often performs just as well for tabular health data while being easily interpretable. Given the number of predictors, Lasso Logistic Regression (LR) would be a natural first choice, with XGBoost serving as a supplementary approach to explore interaction effects. For these reasons, I strongly advise the authors to run and validate Lasso LR along with XGBoost.

**Thank you for this suggestion. We have now run the LASSO logistic regression model using the same train-test splits as for the XGBoost model, with scaled data. This showed that the performance of the LASSO model (AUROC = 0.737 ± 0.059; Figure 1A below) was slightly lower than that of the XGBoost model (AUROC = 0.753 ± 0.058; Figure 1B). While the LASSO model may be easier to interpret, the XGBoost model provides similar information and slightly better performance. For this reason, we present only the results from the XGBoost model in the manuscript. We now further explain our choice to use the XGBoost algorithm in lines 152-157 of the revised manuscript.**

**A**

**B**

***Figure 1. A) Cross-validated performance of the LASSO model during training. B) Cross-validated performance of the XGBoost model during training.***

Secondly, hyperparameter tuning should be reconsidered. The current XGBoost hyperparameter space is dangerously narrow (number of trees: 1–100, learning rates: 2 options, tree depth: 1–2), and expanding it may improve algorithms’ performance and give more robust results. A more suitable range may include 50–1000 trees, learning rate of 0.01–0.3 and tree depth between 2 and 10. In addition, including L1-regularisation (alpha parameter 0 - 10) is recommended given a large number of predictor compared to the sample size.

**Thank you for this comment. We agree that the hyperparameter range used was narrow. This selection was determined following an initial testing of a broader range of parameters. We now specify this in the Materials and Methods section (lines 159-163). As suggested, we have also broadened the range of initially tested hyperparameters to 10-1000 trees, learning rate from 0.01 to 0.3, tree depth between 1 and 10, and alpha from 0 to 10 (Figure 2 below). Following this initial evaluation, we have fine-tuned the model using the following hyperparameter ranges: 10 to 400 trees, learning rate 0.1 or 0.3, tree depth from 1 to 3, and alpha 1 (Figure 1B above). This – especially tuning the alpha parameter – resulted in a slightly improved performance, with an AUROC of 0.753 ± 0.058, compared to 0.751±0.059 previously. We have updated the manuscript accordingly to include these new results (Figure 1; Figure 2; lines 41, 199-200, and 222).**

alpha=1

alpha=10

alpha=0

alpha=0.1

***Figure 2. Initial tuning of the XGBoost model hyperparameters.***

Thirdly, some clarification on Validation Metrics and predictive (ML) approach justification are needed. It appears that AUC and accuracy are reported from the validation metrics, specifically AUC from out-of-sample participants across 10 10-fold cross-validations - could the authors explicitly clarify this in the text.

**Thank you for this comment. We now clarify that the reported AUC represents the out-of-sample cross-validated performance (lines 163-164).**

Further, the decision to take a predictive approach should be better justified. An alternative would be to use multinomial regression or Lasso Logistic Regression to analyze the impact of various exposures on the likelihood of seeking help. In my opinion, both approaches are valid, but the strengths of the chosen predictive methodology should be clearly articulated.

**We now substantiate more extensively our use of the predictive XGBoost approach in lines 144-147 and 152-157. We were interested in both explaining variable associations and evaluating the extent to which help-seeking behaviour could be predicted from available data.**

Finally, representativeness of the sample remains a key concern. I appreciate that this limitation is acknowledged, but I would also note that the sample may be biased toward individuals active on social media, and can be further skewed by the platforms’ algorithms in determining whom to show the ad. Additionally, the country of participants is not reported, which is significant given that healthcare systems and access barriers vary widely. For instance, anecdotal evidence from the UK suggests that seeking professional help often requires framing symptoms in terms of their impact on work and daily responsibilities, as personal pain and discomfort related to menstruation are more easily dismissed.

**Thank you for this comment. We have updated the limitations section to reflect the concerns you have raised issues about the recruitment strategy employed and the potential biases this may introduced (lines 307-316). Further, we have now specified in the abstract and the main text of the manuscript (lines 31 and 92-93) that data was from participants currently residing in the UK.**

To further strengthen the study and future research in this area, I suggest mentioning the importance of inclusion of the socioeconomic indicators along with professional status (even a crude measure of participants’ wealth or socioeconomic status could provide more context for help-seeking behaviors) and engaging PPI (Patient and Public Involvement) Groups in study conceptualisation which could enhance the design and help identify previously missed symptoms, barriers, and their perceived importance.

**Thank you for this suggestion. We have expanded the limitations section to suggest that future work should aim to also include socioeconomic status or deprivation indicators given the impact they may have on access to formal care (lines 320-323). We have also added a recommendation that future works should aim to engage in co-design activities with individuals from the population of interest to ensure that a highly relevant set of variables, from the perspective of those experiencing premenstrual symptoms, is included in the model (lines 323-327).**

In conclusion, this study is well-presented, with a carefully designed and well-described methodology and questionnaires choice and modifications. The topic is socially important, and I encourage improvements in the data analysis to strengthen its potential for publication. Moreover, despite the above-mentioned concerns, given a relatively large sample size and the high prevalence of distressing symptoms such as suicidal thoughts, it would be great to see this study being published once methodological concerns are addressed.

**Thank you for your kind feedback.**
